# Efficient High-Refractive-Index Azobenzene Dendrimers Based on a Hierarchical Supramolecular Approach

**DOI:** 10.1021/acs.chemmater.3c00550

**Published:** 2023-04-20

**Authors:** Sandra Fusco, Stefano Luigi Oscurato, Marcella Salvatore, Francesco Reda, Sara Moujdi, Michael De Oliveira, Antonio Ambrosio, Roberto Centore, Fabio Borbone

**Affiliations:** † Department of Chemical Sciences, University of Napoli Federico II, Complesso Universitario di Monte Sant’Angelo, Via Cintia, 80126 Napoli, Italy; ‡ Department of Physics E. Pancini, University of Napoli Federico II, Complesso Universitario di Monte Sant’Angelo, Via Cintia, 80126 Napoli, Italy; § Centro Servizi Metrologici e tecnologici Avanzati (CeSMA), University of Napoli Federico II, Complesso Universitario di Monte Sant’Angelo, Via Cintia, 80126 Napoli, Italy; ∥ CNST@POLIMI - Fondazione Istituto Italiano di Tecnologia, Via Pascoli 70, 20133 Milano, Italy

## Abstract

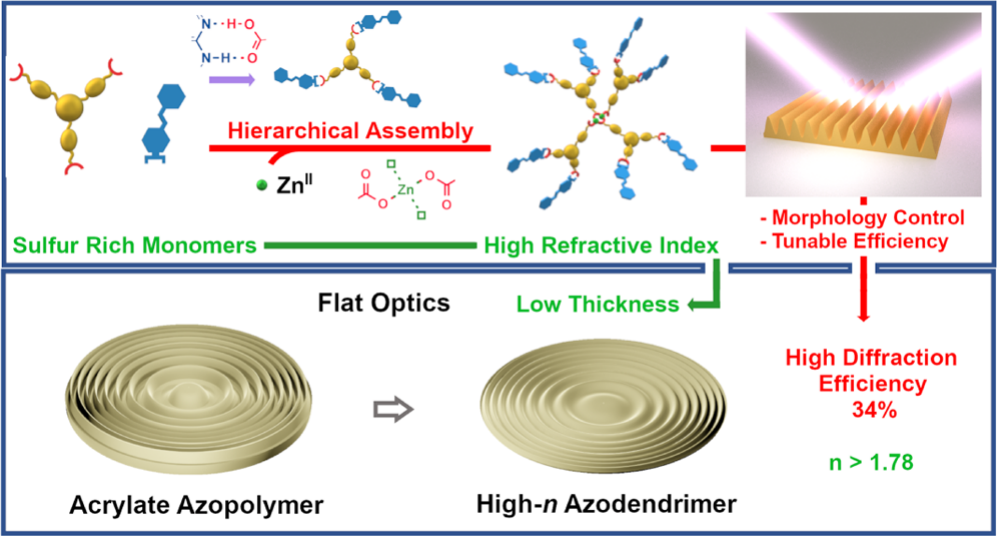

Real-time manipulation of light in a diffractive optical element made with an azomaterial, through the light-induced reconfiguration of its surface based on mass transport, is an ambitious goal that may enable new applications and technologies. The speed and the control over photopatterning/reconfiguration of such devices are critically dependent on the photoresponsiveness of the material to the structuring light pattern and on the required extent of mass transport. In this regard, the higher the refractive index (RI) of the optical medium, the lower the total thickness and inscription time can be. In this work, we explore a flexible design of photopatternable azomaterials based on hierarchically ordered supramolecular interactions, used to construct dendrimer-like structures by mixing specially designed sulfur-rich, high-refractive-index photoactive and photopassive components in solution. We demonstrate that thioglycolic-type carboxylic acid groups can be selectively used as part of a supramolecular synthon based on hydrogen bonding or readily converted to carboxylate and participate in a Zn(II)–carboxylate interaction to modify the structure of the material and fine-tune the quality and efficiency of photoinduced mass transport. Compared with a conventional azopolymer, we demonstrate that it is possible to fabricate high-quality, thinner flat diffractive optical elements to reach the desired diffraction efficiency by increasing the RI of the material, achieved by maximizing the content of high molar refraction groups in the chemical structure of the monomers.

## Introduction

Photoisomerization of azobenzene and its derivatives is a unique and powerful feature as it involves a significant conformational change of this kind of molecules due to the *trans-cis* isomerization, which is accompanied by considerable molecular motion. Functionalization of a material with azobenzenes (“azomaterial”) is the practical way to amplify this light-induced nanoscopic movement to produce macroscopic structural modifications of the material bulk, through collective reorientation of azomolecules sustained by light irradiation.^1^ When an azomaterial film is irradiated with an interference pattern of light, the macroscopic reconfiguration can translate into mass transport occurring on the surface, with the formation of periodic topographic modulations known as surface relief gratings (SRG).^2^ This simple and reversible all-optical process is a unique and intriguing phenomenon and has been object of extended research because of potential applications in numerous fields. While the texturing or reconfiguration of a surface may be exploited to anisotropically control the wettability,^3,4^ to influence the growth of living cells,^5−7^ or in soft lithographic techniques,^8−10^ the spatial modulation of the refractive index, reflecting the modulation of the optical path of an incident light beam in a structured film of a homogeneous azomaterial, is at the core of applicability of SRGs in photonics. Mono- and bidimensional diffractive optical elements (DOE) can be easily fabricated on a large area and with the desired structural complexity to provide different optical functionalities, for applications in spectral filters,^11−13^ light couplers,^14−16^ light-harvesting layers in solar cells,^17,18^ optical outcouplers to improve OLED efficiency,^19,20^ and DFB lasers.^21,22^ Recently, we have demonstrated that a planar DOE can be generated and reshaped in real time into a different one on the same area of an azopolymer film by tuning the surface morphology directly in the light path,^23,24^ a possibility not offered by conventional photolithographic techniques. This approach may enable applications based on the real-time light manipulation through continuous reconfiguration of a DOE. To this aim, highly efficient photoresponsive azomaterials are required to provide rapid rewriting of a surface texture. Additionally, increasing the bulk refractive index of the material could produce the desired diffraction efficiency with a lower SRG amplitude, eventually reducing the exposure time and total thickness of the device. Increasing the refractive index is a sought-after feature to improve properties such as light confinement, propagation efficiency, and acceptance angle for the development of waveguides and components such as sensors or waveguide combiners, the latter being key components of Augmented Reality or Mixed Reality devices.^25,26^ In this work, we address these problems by designing an efficient high-refractive-index azomaterial. One of the best strategies to increase the refractive index of organic materials while maintaining high optical transparency is the introduction of atoms/groups possessing high molar refraction,^27^ as −C–S(II)–C– (7.80) and heteroaromatic units containing −C=N–N– (3.46) and −C=N–C– (4.10) bonds.^28,29^ Polymers with a high content of these groups are reported to show the highest refractive indexes known in the literature (*n* > 1.7),^27^ thus convenient and easily accessible sulfur-functionalized triazines and thiadiazoles are good candidates to maximize the density of these high molar refraction groups and enhance the *n* values of a material. On the other hand, to avoid dilution by low molar refraction alkyl chains, the resulting rigid, heterocycle-rich material structure would not meet the requirements of solubility and responsiveness for a fast SRG inscription process. In this regard, supramolecular chemistry is a powerful, versatile, and well-established tool for overcoming these problems and in general for the design of efficient azomaterials. Particularly, supramolecular azodendrimers, in which azobenzene molecules are linked to the peripheral groups of a dendrimeric oligomer through weak interactions, have proven to be very efficient in terms of SRG formation and to outperform dendrons and dendronized polymers, probably due to their isolated architecture.^30−32^ In this work, we designed a practical and flexible multifunctional dendrimeric supramolecular assembly approach to increase the density of high molar refraction heterocycles in the dendrimer core, without adversely affecting solubility and simultaneously maximizing the efficiency of photoresponsiveness in the resulting material. We have recently demonstrated that the high directional double hydrogen bonding in the 2-aminopyrimidine/carboxylic acid supramolecular synthon is an effective strategy to graft an aminopyrimidine-containing azomolecule to a carboxyl group of a polymer chain to produce stable amorphous azopolymers with enhanced mass transport properties.^33^ On the other hand, metal coordination has been widely demonstrated as a simple and efficient strategy to grow dendritic structures by self-assembly, without the need for purification.^34^ The cooperative action of Zn(II)-carboxylate coordination interactions has been proposed as a straightforward way to tune the properties of a material by simply varying the metal-to-ligand molar ratio.^35^ Thus, our supramolecular design has been inspired by this double functionality of the carboxyl group, which may act as one-half of a hydrogen-bonding-based supramolecular synthon and as a carboxylate in a coordination bond to a metal, giving rise to two types of orthogonal and hierarchically ordered weak interactions, which can be selectively used to change the material structure by effortless tuning of the building blocks composition in solution. Through this approach, high-refractive-index azomaterials were easily prepared and their photoresponsive behavior proved to be readily tunable to optimize the efficiency and quality of the SRG inscription process and to fabricate planar holographic lenses with reduced thickness and optimized diffraction efficiency.

## Results and Discussion

### Material Design and Synthesis

**Figure 1:**
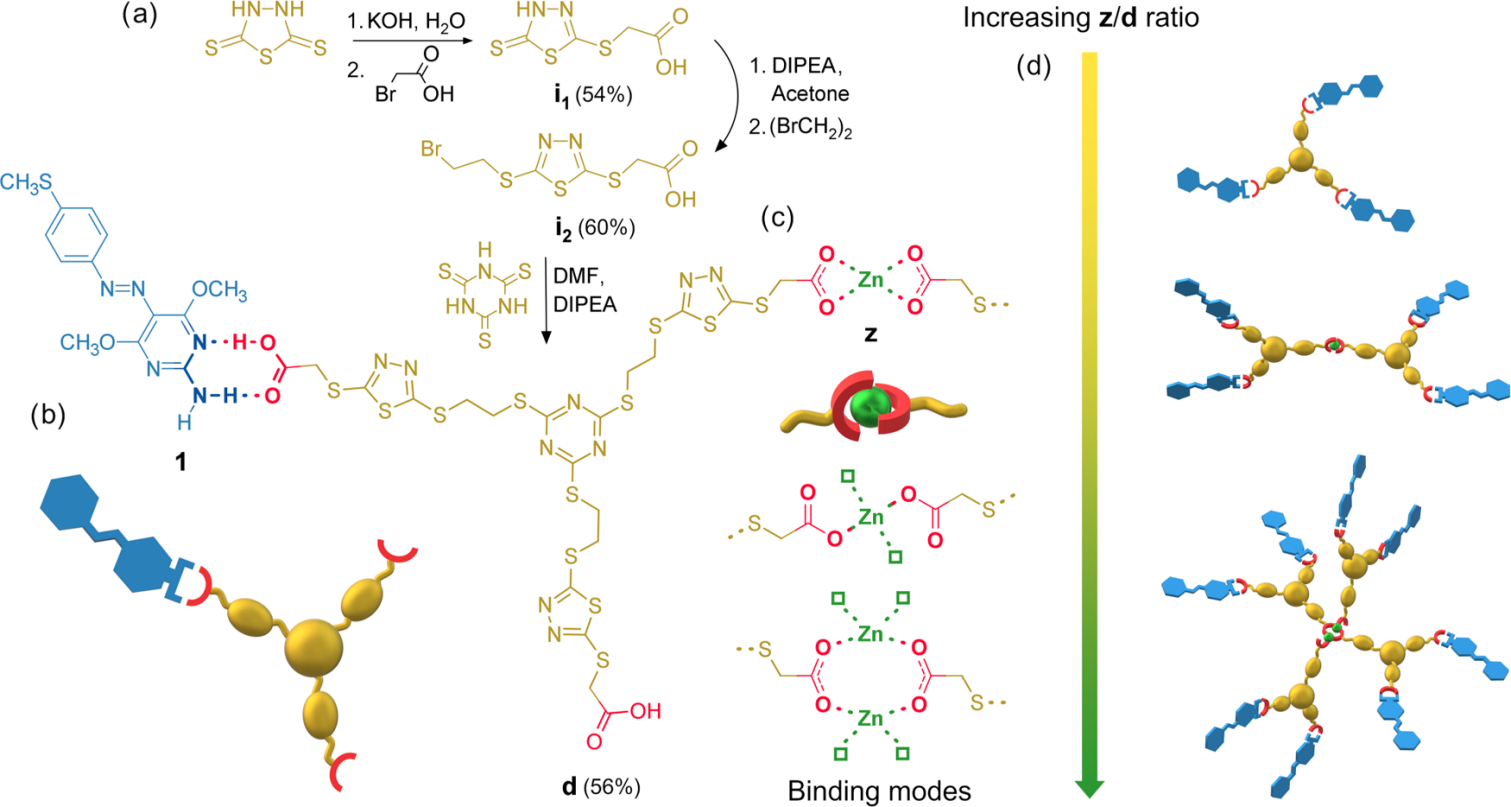
Synthesis scheme of **d** (a); supramolecular synthon between **1** and **d** (b); possible Zn(II)-carboxylate binding modes (c); and scheme of increasing aggregation due to bridging Zn(II)–carboxylate interactions with increasing **z**/**d** ratio (d).

**Figure 2:**
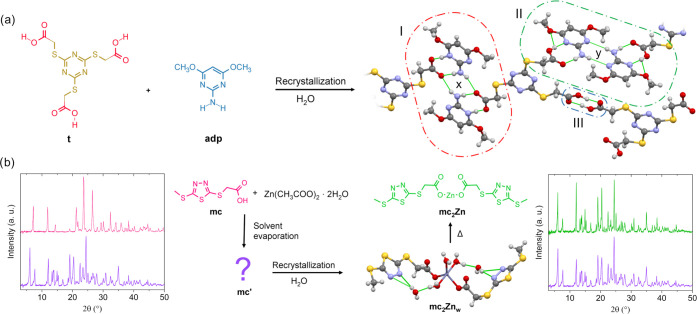
Crystallization scheme of **t(adp)**_**2**_ complex and SCXRD structure (a). PXRD spectra of **mc** (red line) **mc′** (purple line), **mc**_**2**_**Zn** (dehydrated **mc**_**2**_**Zn**_**w**_, green line), and SCXRD structure of **mc**_**2**_**Zn**_**w**_ (b).

The model of the azomaterial is based on a first-generation three-arm dendrimer with thioglycolic-type carboxyl peripheral groups (**d**, Figure 1). Heterocycles containing high-molar-refraction atoms/bonds were selected among sulfur-functionalized triazines and thiadiazoles to build the dendritic core. The optimal synthetic pathway to dendrimer **d** was found by the convergent approach shown in Figure 1. Straightforward sequential functionalization of thiol groups of 1,3,4-thiadiazole-2,5-dithiol with bromoacetic acid and 1,2-dibromoethane led to the intermediate **i**_**2**_ in good yields, then reacted with trithiocyanuric acid to give the final product. The azobenzene structure (**1**, Figure 1) was functionalized with a methylthio group and a 2-aminopyrimidine heterocycle and synthesized according to our recently reported simplified procedure.^33^ Direct alkylation of trithiocyanuric acid with bromoacetic acid afforded the product **t** (Figure 2a) used as a model compound for X-ray study.

Complexation with azomolecules can be realized through the supramolecular heterosynthon based on two hydrogen bonds between the acid and aminopyrimidine groups (AP heterosynthon) shown in Figure 1b, giving rise to an *R*_2_^2^(8) ring pattern. By this strong molecular recognition interaction, the photoresponsive molecules are grafted as termini of the dendritic core. Alternatively, one or more carboxylic functions can be converted to carboxylate and used as focal points of dendrons to build dendrimers with higher molecular weight through Zn(II)–carboxylate coordination interactions (Figure 1c,d). According to the different possible Zn(II)–carboxylate coordination binding modes and to the crystal structures of zinc(II)-carboxylate complexes,^36,37^ increasing contribution by bridging tetra-coordination modes is expected to occur by increasing the ratio between Zn^2+^ and **d**. Therefore, evolution of the material structure toward 1D/2D dendronized coordination polymers or crosslinked systems is expected for high zinc content. However, the SRG inscription efficiency on azomaterials thin films was used as feedback for choosing the appropriate range of compositions to be investigated.

### Model Compounds

The structure of azomolecule **1** was solved by single-crystal X-ray diffraction analysis (SCXRD) and is shown in Figure S1 (crystal data are reported in Table S1). We also investigated by SCXRD the interaction in the supramolecular synthon of thioglycolic peripheral groups with the aminopyrimidine moiety of the azomolecule, after successful cocrystallization of compound **t** with 2-amino-4,6-dimethoxypyrimidine (**adp**) in hot water, which afforded single crystals of the **t(adp)**_**2**_ complex (Figure 2a). In the structure of the complex, each molecule of **t** is coordinated to two **adp** molecules through an AP-type supramolecular heterosynthon, while the third carboxyl is involved in an acid/acid type (AA) homosynthon (Figure 2a_III_). In the AP synthon of Figure 2a_II_, the bond distances and angles of the N–H···O, OH···N, C–O, and C=O bonds are in agreement with typical values (Table S2)^33^ while a more ionic character of the interaction was observed in the case of Figure 2a_I_, where a stronger donation in the OH···N bond occurs, as evidenced by the closer values of C–O and C=O bond distances (1.262 and 1.238 Å, respectively). In case I, the carbonyl and amino groups of two different AP synthons are bridged to form a further ring pattern of hydrogen bonds of the *R*_4_^2^(8) type (*x*), while in case II, a further *R*_2_^2^(8)-type ring due to the PP homosynthon between two **adp** molecules is established (*y*). These situations illustrate two possible ways of supramolecular interactions between different dendrimers in the azomaterials.

Due to the stronger acidity of thioglycolic-type carboxylic group (pK_a_ of thioglycolic acid = 3.55^38,39^) compared to acetic acid (p*K*_a_ = 4.76), easy conversion of dendrimer peripheral groups to Zn(II)-carboxylate can be directly accomplished in situ, during the formulation of the azomaterial in solution, by acid–base reaction with zinc acetate dihydrate. This mechanism, which may lead to an increase of the dendrimer generation, was demonstrated by preparing the zinc salt of the model compound **mc** (Figure 2b). By simple dissolution of **mc** and zinc acetate dihydrate with a 2:1 molar ratio in ethanol and subsequent removal of solvent in vacuo, a white crystalline powder was obtained, **mc′**, showing a different PXRD spectrum than **mc**. This product was recrystallized by slow evaporation of a hot water solution to give large block-shaped single crystals. SCXRD analysis showed the new product to be the hydrated form of the **mc**_**2**_**Zn** salt, **mc**_**2**_**Zn**_**w**_. The complex shows three water molecules coordinated to the metal center and two water molecules as crystallization solvent, which give rise to a bidimensional network of hydrogen bonds (Figure S2). The weight loss in the thermogravimetric diagram is consistent with this structure, showing two steps due to the sequential loss of crystallization and coordinated water molecules (Figure S3). Treatment of **mc**_**2**_**Zn**_**w**_ crystals at 150 °C aimed at removing all of the water content and isolating the **mc**_**2**_**Zn** salt produced a white powder showing the same PXRD pattern as **mc′**, thus proving that the process of mixing **mc** and zinc acetate dihydrate in solution followed by solvent evaporation quantitatively yields the Zn(II)-thioglycolate salt, **mc**_**2**_**Zn**. This mechanism allows the structure and properties of the azomaterial to be easily and hierarchically tuned by simply adjusting the ratio of complementary functions to COOH groups, directly in situ.

### Azomaterials

The azodendrimers **1dz**_**ij**_ were prepared by mixing **1**, **d**, and zinc acetate dihydrate in dimethylformamide (DMF) to have an overall 1:3 azodendrimer/solvent weight ratio. To investigate the effect of the metal concentration on the photoresponsive behavior of the materials, the composition of the building blocks was chosen by setting different i/j molar ratios between **d** and Zn^2+^ (**dz**_**ij**_) and adjusting the azochromophore content to saturate the remaining free COOH groups. Since a highest **dz** ratio than **dz**_**21**_ resulted in unstable solutions and liquid-phase separation, we investigated four compositions below this value, namely, **1d**, **1dz**_**81**_, **1dz**_**41**_, and **1dz**_**21**_. Stable transparent thin films were obtained over the entire range of compositions by spin coating or drop-casting the solutions, followed by treatment under vacuum. The FT-IR analysis shows the shift of the absorption band associated with the carbonyl stretching of the carboxylic group from 1713 cm^–1^ of **d** to 1735 cm^–1^ of **1d**, indicating a change of the interaction pattern from the dimer of the AA homosynthon to that of the AP heterosynthon (Figure S4).^33^ Moreover, the NH_2_ scissoring band of **1** at 1631 cm^–1^ is shifted to a higher frequency (1678 cm^–1^) in **1d**.^40^ This shift increases with the strength and number of hydrogen bonds, and its value is compatible with the double hydrogen-bond patterns described in Figure 2a_I-II_.^33,41^ Formation of zinc carboxylates species could not be observed in the spectra of azodendrimers **1dz**_**ij**_, showing the same spectrum as **1d**, because of signal overlapping in the diagnostic range 1400–1600 cm^–1^. Thus, we recorded the spectrum of **dz**_**21**_ without the azochromophore, showing the expected increase of the band of COO^–^ stretching at 1625 cm^–1^ due to Zn(II)-carboxylate species compared to the spectrum of **d** (Figure S4), while the stretching band of zinc acetate at 1570 cm^–1^ is not visible. The UV–visible absorption spectrum of **1** in DMF shows an intense band at 376 nm due to the π → π* transition of the azobenzene system and a second band at about 450 nm corresponding to the n → π* transition (Figure S5). The spectrum of **d** is shifted toward lower wavelengths and shows a local maximum at 286 nm, so there is no absorption overlap between the photopassive and photoactive components at the wavelengths used for SRG inscription. This separation is also evident in the spectra of **1dz**_**ij**_ as thin films (Figure S6), characterized by the expected broadening and an increasing ratio of the bands at 264 and 382 nm along the **1d** -**1dz**_**21**_ series, reflecting the trend of relative concentration. The refractive index *n* of **1dz**_**ij**_ azomaterials and the acrylic azopolymer **azp** used as comparison model, whose structure is reported in Figure S7,^4,42^ were evaluated by ellipsometry and the values of *n* at the standard wavelength of 633 nm obtained from the dispersion curves reported in Figure S8. The results for **1d** (1.773), **1dz**_**81**_ (1.785), **1dz**_**41**_ (1.783), and **1dz**_**21**_ (1.748) confirm that the optimized design with highly polarizable atoms and groups in the structure of both the azomolecule and the photopassive dendrimer core can produce azomaterials with distinctly high absolute values of the refractive index, even if compared with azopolymers containing higher concentrations of standard fully aromatic azobenzene units (**azp**, 1.691) or even similar aminopyrimidine-based azomolecules (1.638–1.706).^33^

Differential scanning calorimetry (DSC) diagrams of the samples revealed an increase of the glass-transition temperature from 10.0 °C of **1d** to 14.9 °C of **1dz**_**21**_, which can be attributed to the effect of increasing aggregation of first-generation dendrimers induced by Zn(II)–carboxylate interactions (Figure S9). Although the increase of *T*_g_ is limited to only 5 °C, the actual change in material structure occurring in the range of compositions investigated was found to be suitable to define the best compromise between inscription efficiency and stability of induced SRGs, as discussed below.

### SRG and Diffractive Lenses

**Figure 3:**
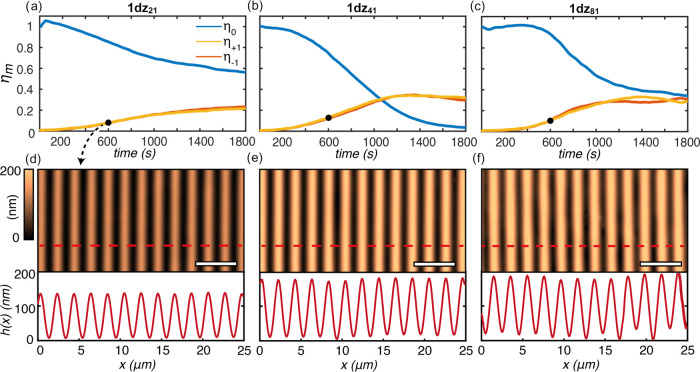
Comparative mass transport dynamics in an SRG inscription experiment. Evolution of the relative diffraction efficiency in the first three diffraction orders of the developing SRG gratings for the **1dz**_**21**_, **1dz**_**41**_, and **1dz**_**81**_ materials (a–c, respectively). Fixed intensity and exposure time of 1800 s have been used. AFM micrographs and topographic cross sections of the SRGs measured for SRGs obtained by 600 s of exposure time (d–f). Both the diffraction efficiency and the topographic analyses highlight increasing mass transport efficiency and increasing profile inhomogeneities from **1dz**_**21**_ to **1dz**_**81**_.

To investigate the relative efficiency and the stability of the mass transport in the **1dz**_**ij**_ azodendrimers, we performed comparative SRG inscription experiments by exposing the films to the sinusoidal intensity pattern of two p-polarized laser beams at λ = 491 nm. According to the scalar diffraction theory, the relative diffraction efficiency η_m_ of the *m*th propagating diffraction order of an ideal sinusoidal surface relief grating at the probe wavelength λ is given by
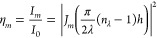
1where *I*_0_ is the total transmitted intensity; *I*_m_ is the intensity of the *m*th diffracted wave; *J*_m_ is the Bessel function of first kind of order *m*; and *h* is the modulation depth of the sinusoidal grating (Figure S10). When applied to the quasi-sinusoidal SRG growing on an azomaterial film during the interferogram irradiation, eq 1 allows a direct connection between the temporal evolution of the recorded diffraction efficiency η_*m*_(*t*) in a specific order and the amplitude *h*(*t*) of the relief grating. The same principle can be applied to directly compare the SRG inscription efficiency of different azomaterials, provided they have a comparable *n*_λ_. In addition, as extensively discussed elsewhere,^43^ eventual discrepancies of the diffraction efficiency of the developing SRG from the predictions of eq 1 can be used to characterize eventual structural deformations of the gratings, providing more elements to evaluate the inscription performances of different azomaterials. In the comparative experiment, we measured the temporal evolution of the diffraction efficiency in the first three orders (*m* = 0, ± 1) for SRGs inscribed on the three **1dz**_**ij**_ azodendrimers, using a He–Ne laser at the wavelength λ_p_ = 633 nm as a probe. The writing intensity and the exposure time has been maintained fixed in the three experiments. The measured diffraction curves are shown in Figure 3a–c. During the SRG inscription experiment, the area of the developing SRGs was additionally irradiated from the substrate side with a collimated circularly polarized laser beam at the λ_a_ = 405 nm. This beam enhances the mass transport and reduces the typical formation of the birefringence grating due to the photoalignment of azomolecules, making the diffraction efficiency dynamics closer to the ideal surface relief grating case of eq 1. Due to the small variations of the refractive index *n*_λ_ for the three materials at the probe wavelength, the diffraction curves in Figure 3 can be directly used to assess the mass transport quality and efficiency. According to eq 1, the initial part of the formation dynamics of a stable quasi-sinusoidal surface relief grating is characterized by monotonic identical increase in the efficiency of ±1 orders, accompanied by a simultaneous decrease in the 0 order. This condition is verified for **1dz**_**21**_ and **1dz**_**41**_, with a clearly slower dynamic in the case of the first azodendrimer. The lower inscription efficiency for **1dz**_**21**_ is further validated by the reduced relief amplitude resulting from AFM analysis of the surfaces at the intermediate exposure time (Figure 3d–f). According to our material design, the azodendrimer **1dz**_**81**_ was expected to show even higher efficiency. However, both the diffraction and the topographic characterizations show that the slight increase in the structuration speed with respect to **1dz**_**41**_ is associated with a reduced stability of the inscription process, with increased structural inhomogeneity in the grating profiles (Figure 3f) and nonideal diffraction behavior (Figure 3c). This instability was even more pronounced in the case of **1d** and was accompanied by a rapid decrease in inscribed SRGs amplitude after the inscription process, which prevented a complete characterization of this material, while the amount of Zn(II)–carboxylate interactions in **1dz**_**81**_ was enough to grant a shelf stability of at least 6 months to the relief amplitude. This trend clearly highlights the flexibility of such materials, where the stability of the inscribed gratings and the photoresponsive behavior can be easily and finely tuned by appropriate balancing of the two hierarchical supramolecular interactions through small changes of the composition of the three molecular building blocks in solution.

**Figure 4:**
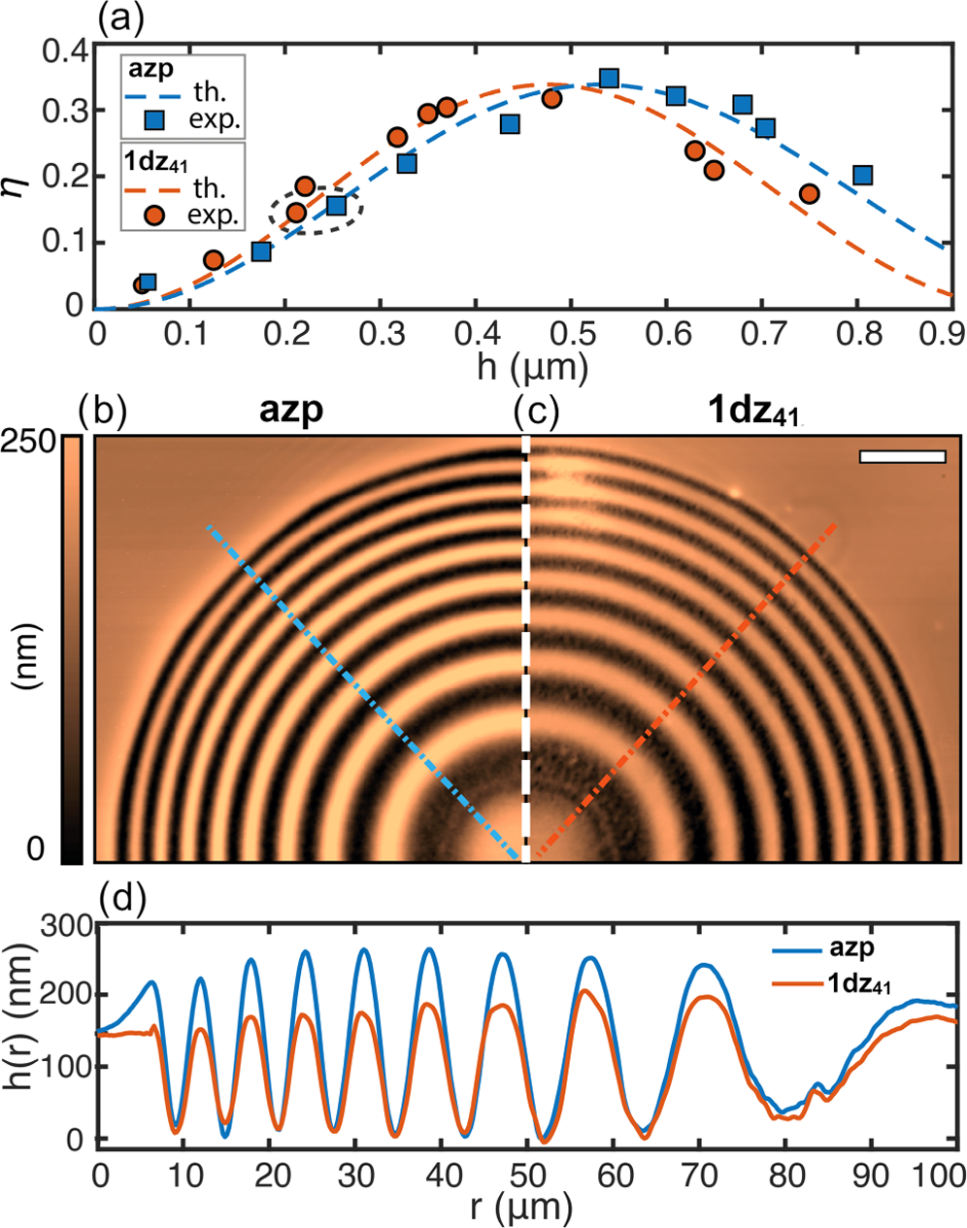
Effect of increased refractive index of the azodendrimers in the fabrication of thin diffractive optical elements. Comparison between the focusing efficiency in the first focal order for a diffractive lens inscribed in the **1dz**_**41**_ azodendrimer (orange circles) and in the acrylic azopolymer (**azp**, blue squares), measured for different average relief amplitude *h* (a). Dashed lines are the prediction of the scalar diffraction theory calculated for *n*_d_ = 1.783 and *n*_p_ = 1.691 at λ = 633 nm. AFM micrographs (b, c) and exemplary radial topographic profiles of the lenses (d) highlighted by the dashed ellipse in (a). The measured average amplitudes, where *h*_d_ = 210 ± 5 nm for **1dz**_**14**_ and *h*_p_ = 255 ± 5 nm for **azp** resulting in a (*h*_p_ – *h*_d_)//*h*_p_ ∼ 18% amplitude reduction.

Another direct consequence of eq 1 is that the diffraction efficiency of a surface diffraction grating, and more in general of a diffractive optical component build as a surface relief pattern, can be increased by a higher refractive index *n*_λ_ for a given relief amplitude *h*. Then, a higher-refractive-index material requires a lower modulation amplitude for the maximum diffraction efficiency, allowing the realization of thinner optical diffractive elements. To further highlight the strength of our material design with respect to this aspect, we fabricate and characterize flat diffractive lenses, selected as a prototype of a general diffractive optical element, on the surface of a **1dz**_**41**_ azodendrimer film, compared with same lenses inscribed in the acrylate azopolymer (**azp**). We choose the **1dz**_**41**_ dendrimer according to good compromise between inscription speed and structural stability in the relief formation performances shown in the previous comparative SRGs analysis. For the fabrication of the diffractive lenses, we used a holo-lithographic method based on the projection of a computer-generated structured light intensity pattern on the surface of the azomaterials, which accordingly develop the surface relief with the same geometry. The description of the lens design can be found in refs (23, 24), while additional details about the optical configuration for the experimental inscription and characterization of the lenses are given in the Experimental Section and in the Supporting Information. In the comparative experiment between **1dz**_**41**_ and **azp**, we measured the diffraction efficiency for λ_p_ = 633 nm in the first focal spot produced by the lenses. Lenses with different relief amplitudes are obtained by varying the exposure time at the writing holographic pattern in different regions of the films. AFM analysis has been used to measure the relief amplitude *h* in each of the inscribed lenses as the average of three radial topographic profiles. Figure 4a shows the comparison of the diffraction efficiency measured as a function of the lens amplitude for the two materials. According to scalar diffraction theory, we included in the plot also the expected diffraction efficiency behavior calculated using the refractive indices obtained by ellipsometry for the two materials at the probe wavelength. The two sets of experimental data closely follow the theoretical curves. If, on the one hand, this additionally sustains the accuracy of the measured refractive index by ellipsometry, on the other hand, it allows us to use the theoretical curves to characterize the effect of the refractive index on the performance of the diffractive components. According to Figure 4a, the maximum diffraction efficiency of η ≈ 0.34 is obtained for a relief amplitude of *h*_d_ = 475 nm for the dendrimeric material and for *h*_p_ = 529 nm for the polymer, requiring then 10% thinner amplitude for a **1dz**_**41**_ relief with optimized efficiency. Figure 4b compares the AFM micrographs of the lenses of the two materials having an experimental comparable diffraction efficiency of η ∼ 0.15 ± 0.05 (highlighted by the dashed circle in Figure 4a). As visible from the radial topographic profiles shown in Figure 4c, the lens on **1dz**_**41**_ is characterized by an ∼18% reduction in the average modulation amplitude arising from the increase in the refractive index.

## Conclusions

Here, we present efficient azomaterials with a high refractive index obtained through a flexible design based on hierarchical hydrogen bonding and Zn(II)–carboxylate supramolecular interactions. High refractive index values were achieved by maximizing the density of high molar refraction groups as −C–S(II)–C–, −C=N–N–, and −C=N–C– in the structure of both the photoactive azomolecule and the dendrimer-shaped photopassive component, with almost 0.1 increase compared to conventional acrylate azopolymer and supramolecular polymers with similar azobenzene units. Aggregation and growth of hydrogen-bonded azodendrimers through Zn(II)–carboxylate interactions can be readily induced by the addition of zinc acetate to the formulation and consequent complete anion exchange with thioglycolate-type dendrimer termini. By changing the zinc content, it was possible to finely modulate and balance the efficiency and quality of mass transport during the SRG inscription process, until a theory-matching response of the material. We demonstrated that a thinner holographic lens with the same diffraction efficiency can be fabricated on a thin film of the most performing **1dz**_**41**_ material compared to the standard acrylate polymer **azp**, as a result of the increased refractive index. The combination of high refractive index and high inscription efficiency and quality, enabled by flexible supramolecular chemistry and design, is a key factor in the development of real-time reconfigurable planar photonic devices.

## Experimental Section

All of the reagents were commercially available and used without further purification. ^1^H and ^13^C NMR spectra were recorded on Varian Inova 500 (500 MHz) and Bruker Ascend 400 (400 MHz) spectrometers. DSC diagrams were recorded on a METTLER TOLEDO DSC3+ calorimeter. UV–visible spectra were recorded with a JASCO V-750 spectrometer. The FT-IR measurements were performed on a Thermo Nicolet 5700 FT-IR spectrometer. Solid samples were dispersed in KBr tablets, azomaterials were cast from solution. Ellipsometry data were collected using a commercially available M-2000 Spectroscopic Ellipsometer (J. A. Woollam). Data analysis was performed using Complete EASE software (version 6.57). Topographic analysis of the structured surfaces was performed by an AFM (WITEC α RS300) operating in tapping mode with a cantilever of 75 kHz resonance frequency and spring constant of 3 N/m. Analysis and elaboration of AFM data were accomplished by means of the open-source software Gwyddion. Thin films of azomaterials were prepared by spin-coating DMF solutions on glass substrates, using a Laurell WS-650Mz-23NPP spin coater.

### X-ray Diffraction Analysis

PXRD spectra were recorded on a Panalytical Empyrean multipurpose X-ray diffractometer. Selected crystals of **1**, **mc**_**2**_**Zn**_**w**_, and **t(adp)**_**2**_ were mounted on a Bruker-Nonius KappaCCD diffractometer equipped with Oxford Cryostream apparatus (graphite monochromated Mo Kα radiation, λ = 0.71073 Å, CCD rotation images, thick slices, φ and ω scans to fill asymmetric unit). Reduction of data and semiempirical absorption correction were done using SADABS program. The structures were solved by direct methods (SIR97 program)^44^ and refined by the full-matrix least-squares method on *F*^2^ using SHELXL-2016 program^45^ with the aid of the program Olex2.^46^ H atoms bonded to C were generated stereochemically and refined by the riding model, those bonded to O and N were found in difference Fourier maps and their coordinates were refined. For all H atoms, *U*_iso_(H) equal to 1.2 U_eq_ or 1.5 U_eq_ (C_methyl_) of the carrier atom was used. Crystal data and structure refinement details are reported in Table S1. The figures were generated using Mercury CSD 3.3.^47^ All crystal data were deposited at Cambridge Crystallographic Data Centre with assigned numbers CCDC 2234125 (**1**), 2234126 (**t(adp)**_**2**_), and 2234127 (**mc**_**2**_**Zn**_**w**_). These data can be obtained free of charge from www.ccdc.cam.ac.uk/data_request/cif.

### Synthesis

#### Synthesis of Diazonium Salt of 4-(Methylthio)aniline (**md**)

4-(Methylthio)aniline (4,00 g, 28.7 mmol) and 10.5 g of tetrafluoroboric acid solution 48% in H_2_O were added to 25 mL of water. Sodium nitrite (1.98 g, 28.7 mmol) was slowly added to the mixture at 0–5 °C under stirring. After 30 min, the tetrafluoroborate salt was filtered, washed with cold water and ethyl ether, and dried. Yield: 5.77 g, 84.4%.

#### Synthesis of **1**

2-Amino-4,6-dimethoxypyrimidine (3.76 g, 24.2 mmol) was dissolved in 1,2-dichloroethane (70 mL). **md** (5.77 g, 24.2 mmol) was slowly added under stirring at room temperature. After 24 h, the solvent was evaporated in vacuo and the solid was recrystallized in ethanol/water in the presence of triethylamine. The filtered and dried product was purified by Soxhlet extraction in heptane, giving reddish plate crystals of **1**. Yield: 42%. ^1^H NMR (DMSO-*d*_6_) δ 2.52 (s, 3H), 3.92 (s, 6H), 7.33 (m, 4H), 7.58 (d, 2H). ^13^C NMR (DMSO-*d*_6_) δ 14.57, 53.91, 112.96, 121.90, 125.94, 139.52, 151.14, 161.24, 164.35. Single crystals of **1** were grown by slow evaporation of acetone solution and used for SCXRD analysis.

#### Synthesis of **i**_**1**_

Bromoacetic acid (1.85 g, 13.3 mmol) was added to a solution of 1,3,4-thiadiazole-2,5-dithiol (2.00 g, 13.3 mmol) and KOH 85% (1.76 g, 26.6 mmol) in water (20 mL) under stirring. After 2 h, a white solid was filtered and dissolved in water (25 mL) with gentle warming. The pH was adjusted to 6 with KOH, and the solution was poured into concentrated HCl (50 mL) under stirring. After a few minutes, a pale yellow solid crystallized. After cooling, the product was filtered under vacuum. Yield: 1.50 g, 54%. ^1^H NMR (DMSO-*d*_6_) δ 4.04 (s, 2H). ^13^C NMR (DMSO-*d*_6_) δ 35.10,157.42, 169.10, 188.20.

#### Synthesis of **i**_**2**_

1,2-Dibromoethane (6.83 g, 36.4 mmol) was added to a solution of **i**_**1**_ (1,50 g, 7.20 mmol) and DIPEA (0.931 g, 7.20 mmol) in 15 mL of acetone. After 24 h, the solvent was removed under vacuum and the residue was dissolved in 100 mL of chloroform. The organic phase was washed with water containing HCl. After anhydrification and evaporation of the solvent, the solid was recrystallized in acetone/hexane to give pale yellow crystals of the product (1.35 g yield 60%). ^1^H NMR (Acetone-*d*_6_) δ 3.80 (m, 4H), 4.21 (s, 2H). ^13^C NMR (DMSO-*d*_6_) δ 30.95, 35.34, 35.79, 164.12, 164.89, 169.10.

#### Synthesis of **d**

**i**_**2**_ (3.42 g, 10.8 mmol) and trithiocyanuric acid (0.641 g, 3.60 mmol) were dissolved in 6 mL of DMF under stirring. DIPEA (1.46 g, 11.3 mmol) was added, and the reaction was stirred for 48 h. The product was precipitated into a mixture of 10 mL of water and 15 mL of concentrated HCl as a sticky solid on the bottom of the beaker. After removal of the supernatant, the solid was dissolved in 30 mL of acetone and slowly precipitated into a mixture of 70 mL of water and 30 mL of concentrated HCl to remove residual DMF. The isolated solid was dried, treated with chloroform under stirring, and filtered in vacuo. Finally, the product was dissolved in the minimum amount of acetone, filtered, and isolated by solvent evaporation as a yellowish powder (yield 56%). ^1^H NMR (DMSO-*d*_6_) δ 3.53 (m, 12H), 4.06 (s, 6H). ^13^C NMR (DMSO-*d*_6_) δ 29.42, 33.37, 35.76, 164.45, 164.75, 169.10, 178.50.

#### Synthesis of **mc**

Bromoacetic acid (2,80 g, 20.2 mmol) was added to a solution of 5-methylthio-1,3,4-thiadiazole-2-thiol (3.31 g, 20.2 mmol) and NaOH (1.62 g, 40.4 mmol) in 40 mL of ethanol under stirring. After 1 h, water (2 mL) was added to the white suspension and the mixture was stirred for 24 h at room temperature. The solvent was evaporated in vacuo, the residue was dissolved in water (20 mL) and precipitated in 20 mL of concentrated HCl. The product was washed with water and recrystallized from ethanol (yield 77%). ^1^H NMR (DMSO-*d*_6_) δ 2.73 (s, 3H), 4.13 (s, 2H). ^13^C NMR (DMSO-*d*_6_) δ 16.55, 35.83, 163.69, 167.10, 169.14.

#### Synthesis of **mc**_**2**_**Zn**_**w**_

A mixture of **mc** (0.222 g, 1.00 mmol) and zinc acetate dihydrate (0.110 g, 0.500 mmol) was dissolved in 30 mL of ethanol. After 10 min, the solvent was removed in vacuo to give a white crystalline solid. The solid was dissolved in 10 mL of boiling water, then the solution was left at room temperature. After solvent evaporation, large plate crystals formed, suitable for SCXRD analysis.

#### Synthesis of **t(adp)**_**2**_

Compound **t** was prepared according to a literature procedure.^48^ A mixture of **t** (35.0 mg, 0.100 mmol) and 2-amino-4,6-dimethoxypyrimidine (46.5 mg, 0.300 mmol) was dissolved in 2 mL of boiling water, and the solution was kept at 80 °C for 24 h, after which needle-like crystals of **t(adp)**_**2**_ were obtained, suitable for SCXRD analysis.

### SRG Inscription and Monitoring

The experimental configuration used for the inscription of SRGs on the azomaterial films consisted of two coplanar p-polarized laser beams (Cobolt Calypso, at wavelength λ_w_ = 491 nm), interfering with the sample surface (Figure S11). The sinusoidal intensity fringes had a periodicity of Λ ≈ 2.0 μm, obtained by tuning, symmetrically with respect to the surface normal, the incidence angle of the two beams according to Bragg’s law. The exposure time and the irradiation intensity were kept fixed at 30 min and 230 mW/cm^2^, respectively, for all tested materials. The power and the diameter of each interfering beam were *P*∼17.0 mW and *D*∼4.5 mm, respectively. A homogeneous collimated assisting laser beam (at λ_a_ = 405 nm) was included in the inscription process, impinging on the sample from the substrate side. Circular polarization and intensity ≈70 mW/cm^2^ were used for this beam in all of the SRG inscription experiments. The dynamics of surface structuration for the different materials were investigated by recording the time-evolving relative diffraction efficiencies in the 0 and ±1 diffraction orders of a He–Ne probe beam at λ_p_ = 633 nm (TE polarized), incident on the growing SRGs.

### Diffractive Lens Inscription and Characterization

To inscribe the surface profile of the cosinusoidal Phase Zone Plates (c-PZPs), acting as multifocal diffractive lenses,^24^ the writing laser beam at λ_w_ was phase-modulated by a reflective Spatial Light Modulator (SLM), (Holoeye, Pluto) in a Computer-Generated Hologram (CGH) configuration to generate the corresponding structured intensity pattern in the sample plane (Figure S12). To this aim, the modulated beam is first propagated through a 4f lens system, with the input plane located in the SLM plane. The 4f output plane coincided with the back focal plane of an infinity-corrected long-working-distance 50× objective (Mitutoyo), having numerical aperture NA = 0.55 and WD = 13 mm working distance, focusing the holographic intensity pattern on the surface of the tested materials. The writing beam was circularly polarized by means of a quarter wave plate placed before the objective, while its intensity at the sample plane was ≈14.0 W/cm^2^. The assisting beam at λ_a_, with intensity ≈0.5 W/cm^2^, was used also in this configuration to enhance the surface structuring process. The exposure time interval was chosen based on previous experiments on the azopolymer structuration in similar conditions.^23^ To fully explore the expected focusing efficiency dynamics, an interval of 5–60 s was used to inscribe c-PZPs with focal length *f* = 0.8 mm at λ_p_ = 633 nm, reaching modulation amplitudes in the range 100–800 nm for the tested materials. The relative diffraction efficiency in the first focus of the c-PZPS was measured using the probe beam at λ_p_ orthogonally incident on the diffractive lens from the substrate side, following the procedure extensively described in previous works.^24^

## Supporting Information Available

Crystal packing views of **1** and **mc**_**2**_**Zn**_**w**_; crystal, collection, and refinement data; relevant bond lengths and angles; thermogravimetric curves; FT-IR and UV–visible spectra of molecules and azomaterials; chemical structure of **azp**; refractive index dispersion curves and DSC diagrams of azomaterials; plot of theoretical diffraction efficiency for an ideal SRG; experimental setup scheme for SRG inscription and holographic structuration; and ^1^H and ^13^C NMR spectra of compounds (PDF)

## Accession Codes

CCDC 2234125 (**1**), 2234126 (**t(adp)**_**2**_), and 2234127 (**mc**_**2**_**Zn**_**w**_) contain the supplementary crystallographic data for this paper. These data can be obtained free of charge via www.ccdc.cam.ac.uk/data_request/cif, by emailing data_request@ccdc.cam.ac.uk, or by contacting The Cambridge Crystallographic Data Centre, 12 Union Road, Cambridge CB2 1EZ, UK; fax: +44 1223 336033.
