# Supplementary material for: Efficient High-Refractive-Index Azobenzene Dendrimers Based on a Hierarchical Supramolecular Approach
Source: Chem Mater. 2023 Apr 20;35(9):3722–30. doi: 10.1021/acs.chemmater.3c00550 (PMC10173454; doi:10.1021/acs.chemmater.3c00550)
Supplement: Supplementary file 1 — cm3c00550_si_001.pdf [file cm3c00550_si_001.pdf]

Supporting Information

Efficient High-Refractive-Index Azobenzene Dendrimers Based on a Hierarchical Supramolecular Approach

Sandra Fusco, [a] *Stefano Luigi Oscurato,*[b] *Marcella Salvatore,*[c] *Francesco Reda,* [b] *Sara Moujdi,*[d]

Michael De Oliveira,[d] *Antonio Ambrosio,*[d] *Roberto Centore*[a] *and Fabio Borbone**[a,d]

[a] Department of Chemical Sciences, University of Napoli Federico II, Complesso Universitario di Monte Sant'Angelo, Via Cintia, 80126 Napoli, Italy

[b] Department of Physics E. Pancini, University of Napoli Federico II, Complesso Universitario di Monte Sant'Angelo, Via Cintia, 80126 Napoli, Italy

[c] Centro Servizi Metrologici e tecnologici Avanzati (CeSMA), University of Napoli Federico II, Complesso Universitario di Monte Sant'Angelo, Via Cintia, 80126, Napoli, Italy.

[d] CNST@POLIMI - Fondazione Istituto Italiano di Tecnologia, Via Pascoli 70, 20133, Milano, Italy

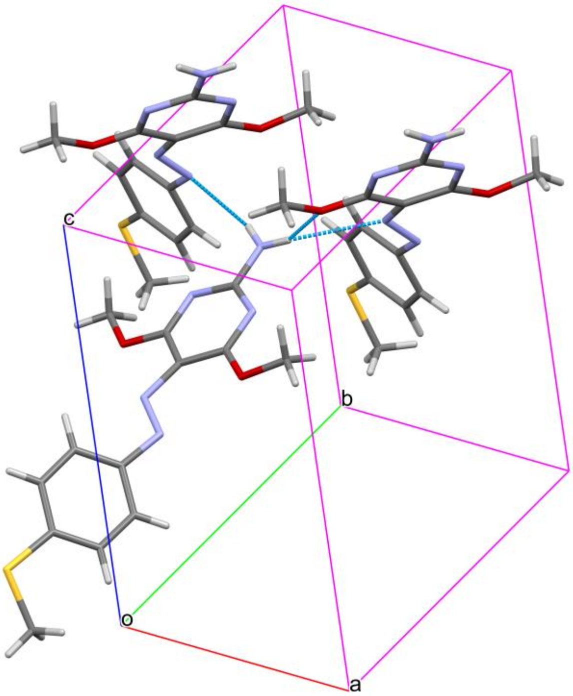

Figure S1 – Partial crystal packing of **1** with highlighted hydrogen bonds.

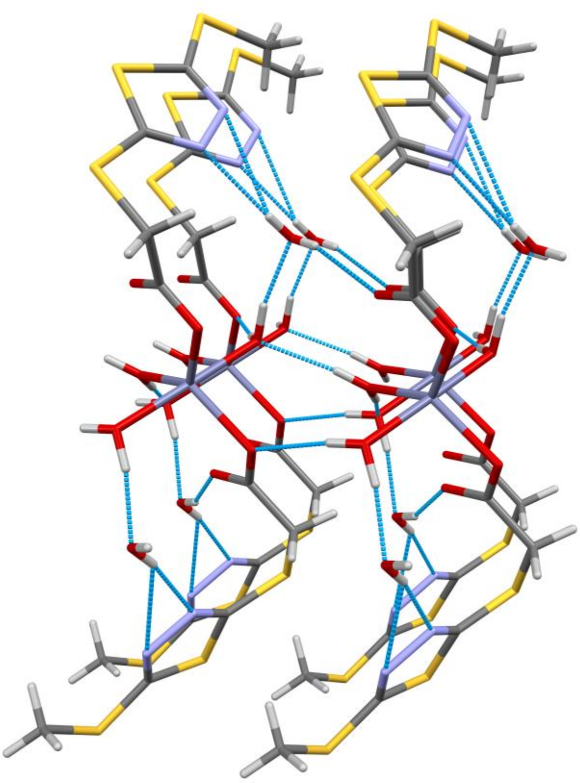

Figure S2 – Partial crystal packing of **mc_2Zn_w** showing the portion of a layer with bidimensional network of hydrogen bonds.

Table S1 Crystal data and structure refinement details for **1**, **t(adp)$_2$** and **mc$_2$Zn$_w$**.

	**1**	**t(adp)$_2$**	**mc$_2$Zn$_w$**
CCDC number	2234125	2234126	2234127
Empirical formula	$C_{13}H_{15}N_5O_2S$	$C_{21}H_{27}N_9O_{10}S_3$	$C_{10}H_{20}N_4O_9S_6Zn$
Formula weight	305.36	661.69	598.03
T (K)	293(2)	293(2)	173(2)
λ (Å)	0.71073	0.71073	0.71073
Crystal system	Monoclinic	Triclinic	Tetragonal
Space group	*P2$_1$/c*	*P-1*	*P4$_1$2$_1$2*
a (Å)	6.524(2)	4.9860(3)	5.423(4)
b (Å)	21.701(5)	10.8500(16)	5.423(8)
c (Å)	11.253(4)	27.126(2)	74.528(14)
α (°)	90	97.117(9)	90
β (°)	109.93(2)	92.981(8)	90
γ (°)	90	96.637(13)	90
V (Å^3)	1497.8(8)	1443.1(3)	2192(3)
Z	4	2	4
D_{calc} (Mg/m^3)	1.354	1.523	1.812
μ (mm^{-1})	0.228	0.326	1.743
F(000)	640.0	688.0	1224.0
θ range (°)	2.69 – 27.50	2.77 - 27.50	3.28 – 27.45
Reflections collected / unique [R(int)]	8923/3290 [0.0272]	11949/6302 [0.0325]	8639/2364 [0 .0364]
Data/restraints/parameters	3290/20/232	6302/2/413	2364/1/155
Goodness-of-fit on F^2	1.044	1.029	1.196
Final *R1*, *wR2* indices [I>2σ(I)]	0.0408, 0.0944	0.0469, 0.0976	0.0355, 0.0761
Final *R1*, *wR2* indices (all data)	0.0701, 0.1089	0.0918, 0.1141	0.0431, 0.0780
Largest diff. peak / hole (eA^{-3})	0.20/-0.21	0.26/-0.25	0.42/-0.37

Table S2 Bond length distances (Å) and angles (°).

**Type I heterosynthon**		
**N_{1B}**-H_{1BA}···**O_{3A}**<br>**2.811**	O_{4A}···$H_{3B}N_{3B}$<br>2.617	C_{7A}-O_{4A}<br>1.262
N_{1B}-**H_{1BA}···O_{3A}**<br>**1.894**	O_{4A}···$H_{3B}N_{3B}$<br>1.711	C_{7A}-O_{3A}<br>1.238
N_{1B}-H_{1BA}···O_{3A} angle<br>178.17	O_{4A}···$H_{3B}N_{3B}$ angle<br>178.26	
**Type II heterosynthon**		
**N_{1C}**-H_{1CA}···**O_{1A}**<br>**2.851**	$O_{2A}H_{2A}$···N_{3C}<br>2.612	C_{5A}-O_{2A}<br>1.293
N_{1C}-**H_{1CA}···O_{1A}**<br>**1.967**	$O_{2A}H_{2A}$···N_{3C}<br>1.621	C_{5A}-O_{1A}<br>1.214
N_{1C}-H_{1CA}···O_{1A} angle<br>172.44	$O_{2A}H_{2A}$···N_{3C} angle<br>178.79	

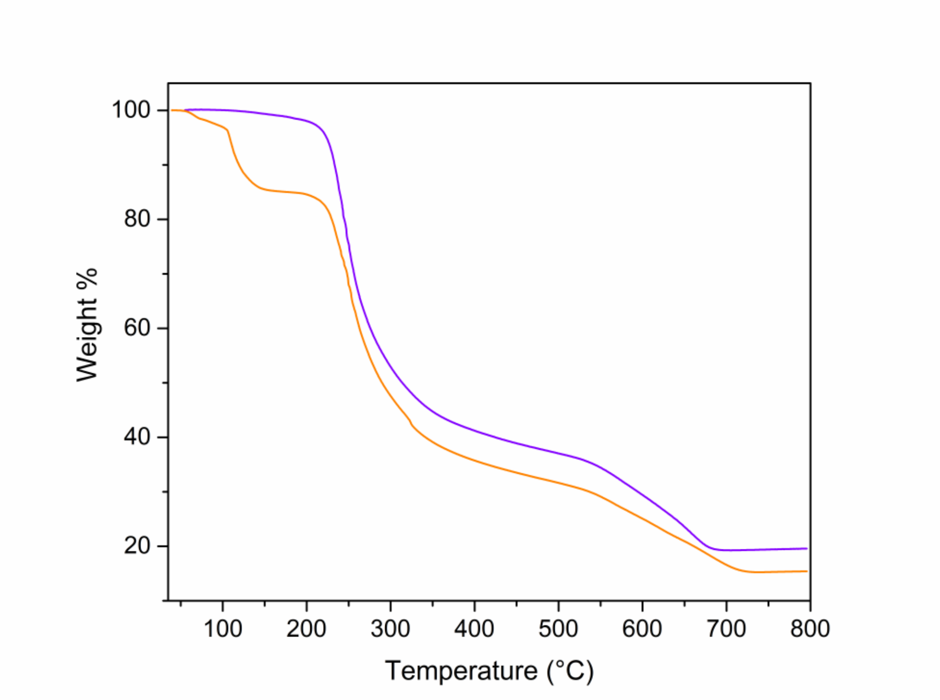

Figure S3 – Thermogravimetric curves of **mc′** (purple line) and **mc_2Zn_w** (orange line).

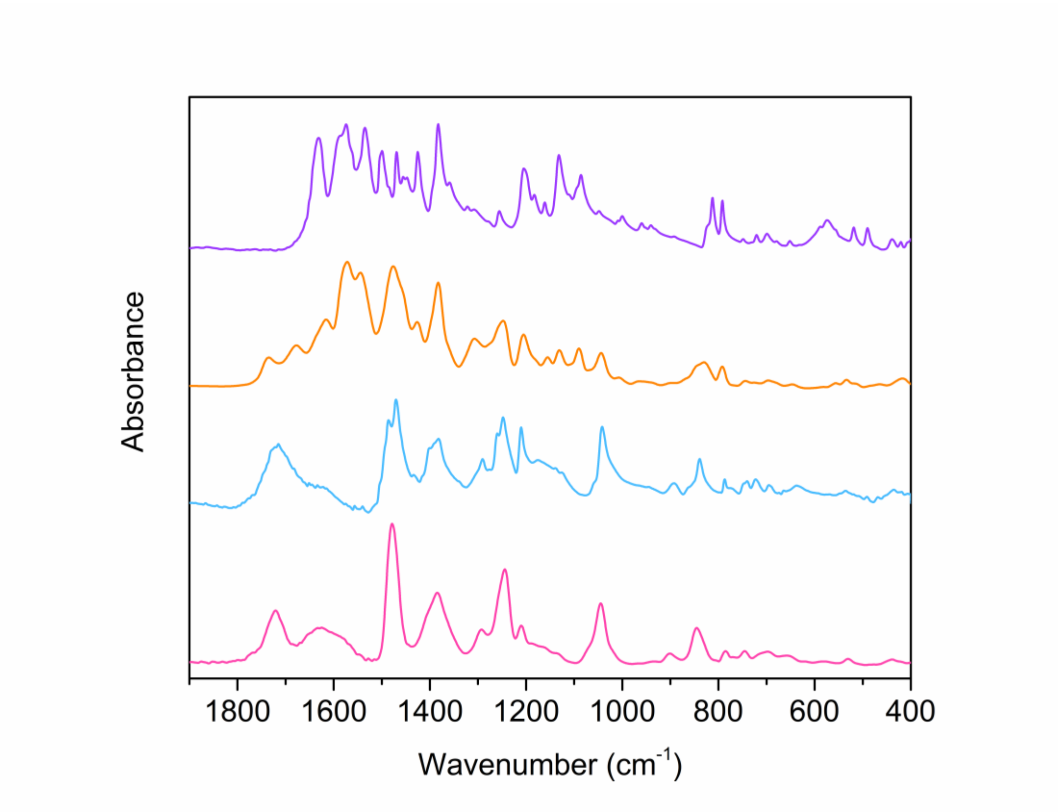

Figure S4 – FT-IR spectra of **1** (purple line), **1d** (orange line), **d** (blue line) and **dz$_{21}$** (magenta line).

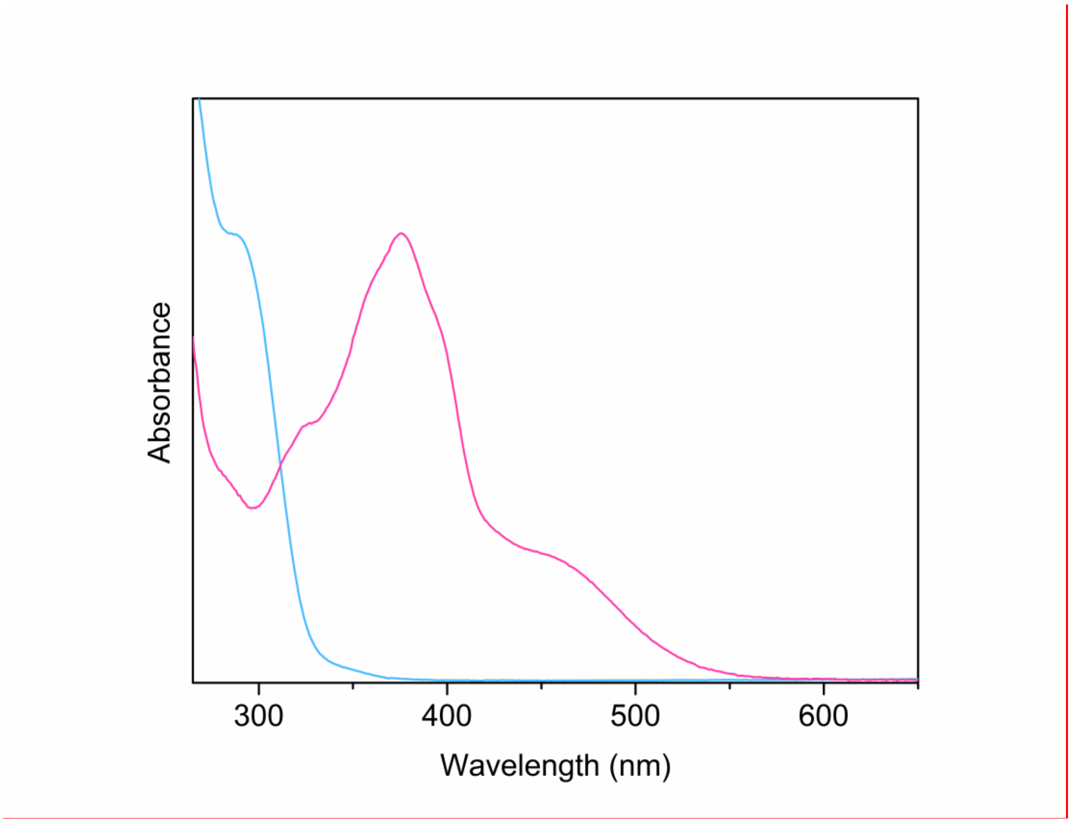

Figure S5 – UV-Visible spectra of **1** (red line) and **d** (blue line) in DMF.

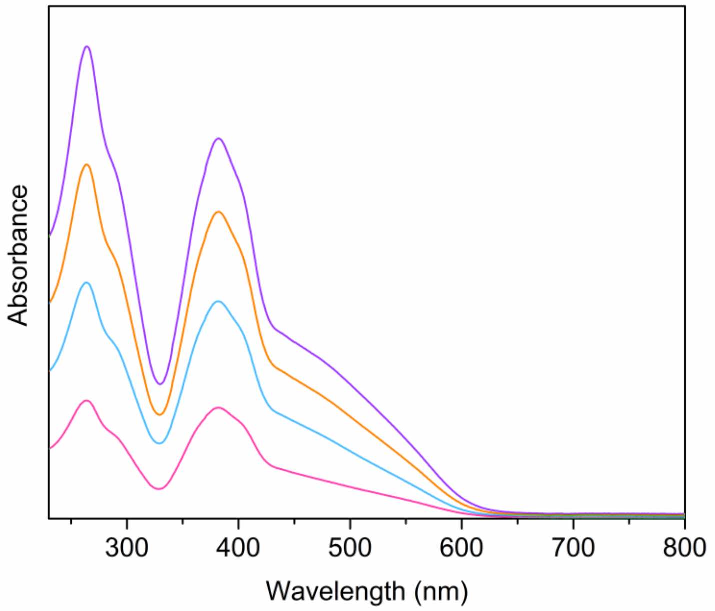

Figure S6 – UV-Visible spectra of **1d** (red line), **1dz$_{81}$** (blue line), **1dz$_{41}$** (orange line) and **1dz$_{21}$** (purple line) as thin films.

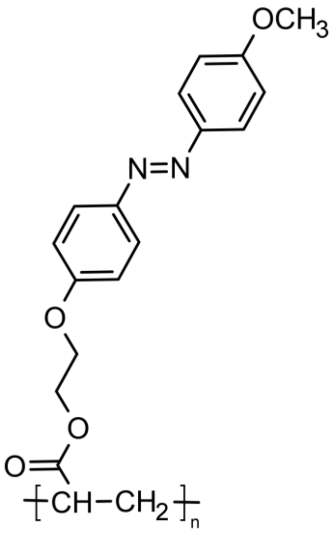

Figure S7 – Chemical structure of polymer **azp**.

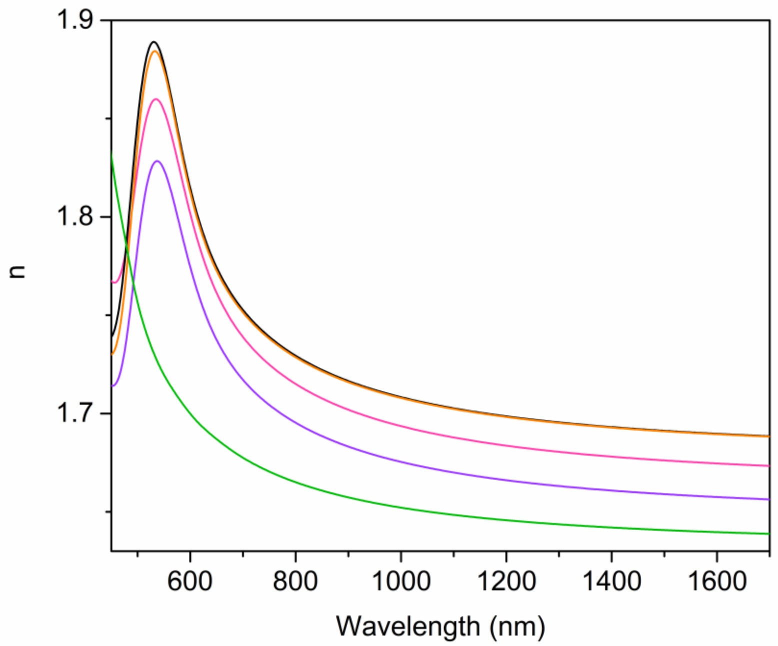

Figure S8 – Refractive index dispersion of **1dz$_{81}$** (blue line), **1dz$_{41}$** (orange line), **1d** (red line), **1dz$_{21}$** (purple line) and **azp** (green line).

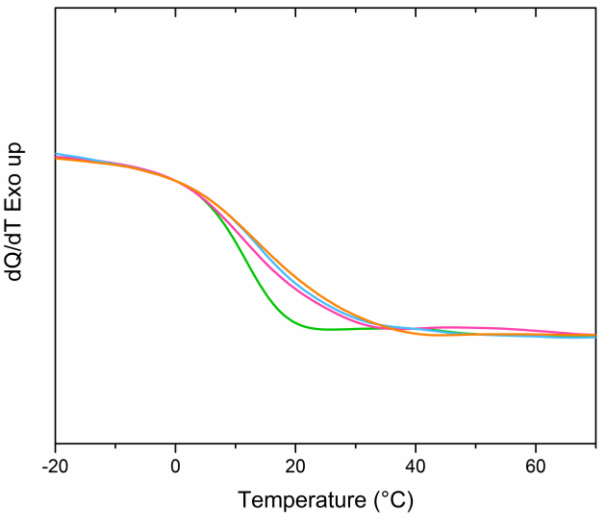

Figure S9 – DSC curves of **1d** (green line), **1dz$_{81}$** (red line), **1dz$_{41}$** (blue line) and **1dz$_{21}$** (orange line).

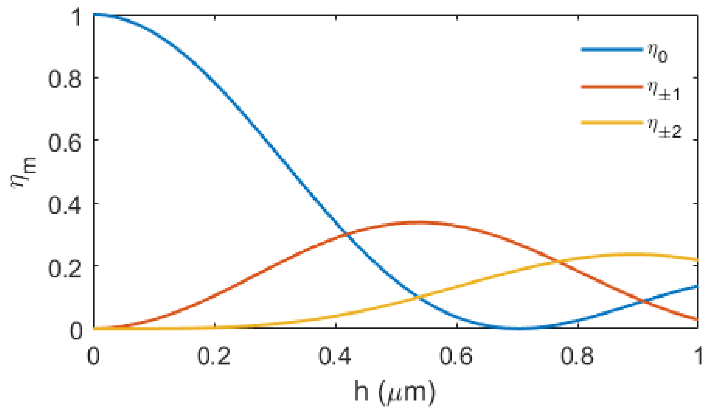

Figure S10 – Diffraction efficiency of the first five diffraction orders plotted with respect to the amplitude modulation h of an ideal sinusoidal surface relief grating. Refractive index n=1.69 and probe wavelength λ=633 nm are assumed for the plot.

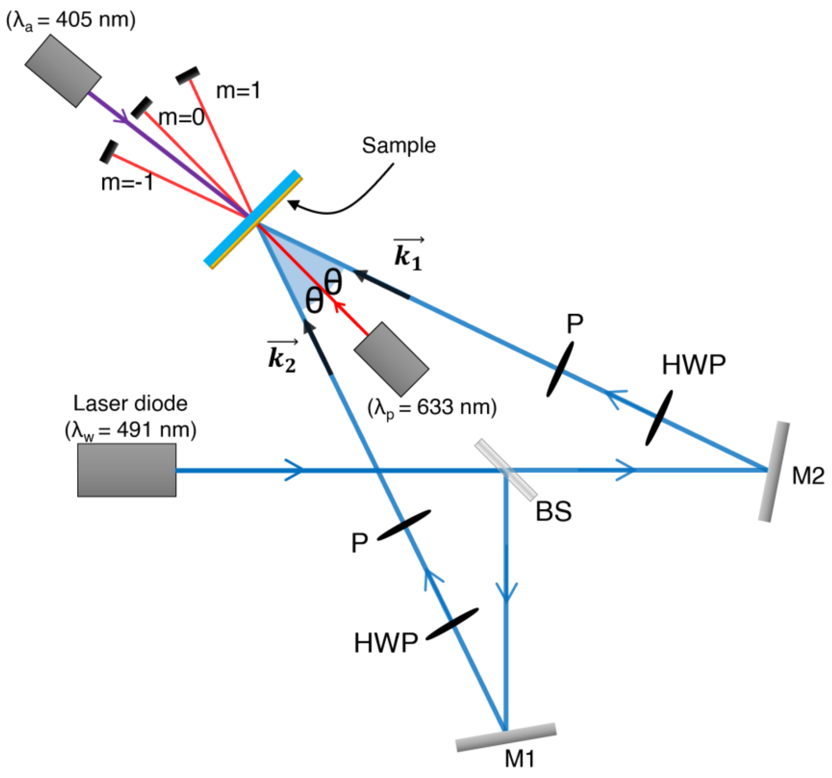

Figure S11 – Experimental setup for the inscription of SRGs. A λ_w=491 nm laser beam is splitted by means of a 50:50 Beam Splitter (BS). Two mirrors (M_1 and M_2) allow to control the angle between the two interfering beams over the material surface. The optical power of each beam is independently tuned by a Half Wave Plate (HWP) and a linear Polarizer (P) in order to maximize fringes visibility in the interferogram. A He-Ne laser beam (λ_p=633 nm) is used for diffraction efficiency measurements. The assisting beam at λ_a=405 nm is circularly polarized before being redirected in the writing area at near-normal incidence.

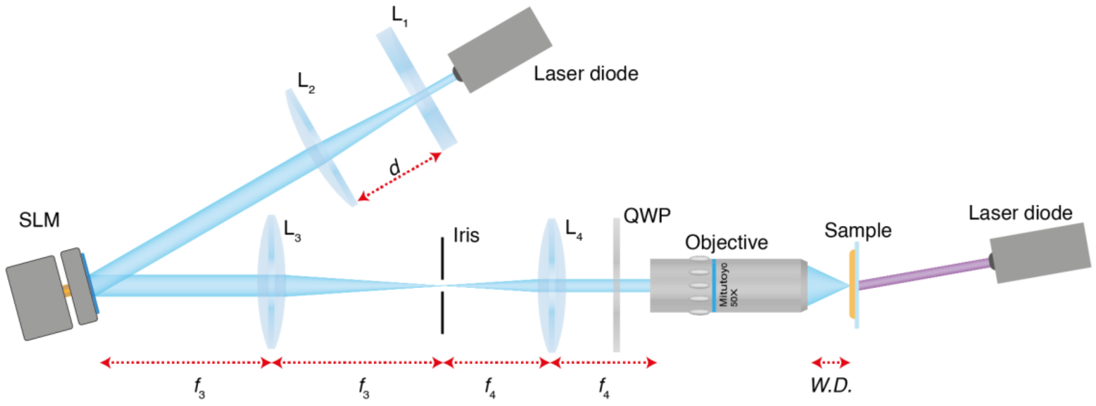

Figure S12 – Experimental setup for holographic structuration of the materials surface. The writing laser beam, at wavelength λ_w=491 nm, after a beam expander (lenses L_1 and L_2), is phase-modulated by a reflective phase-only Holoeye Pluto Spatial Light Modulator (SLM). The SLM realizes a 4*f* configuration with a convex lens (L_3), with a focal length of f_3=300 mm and a convex lens L_4, with focal length f_4=175 mm. An iris is used to filter out all the undesired orders from the SLM. The laser beam is then projected in the back focal plane of an infinity corrected "50X Mitutoyo" objective (OBJ) after imposing circular polarization by means of a quarter wave plate (QWP). The structured holographic intensity distribution is then reconstructed and focused on the objective focal plane (objective working distance WD 13mm) where the azopolymer film is placed. The assisting beam at λ_a=405 nm is circularly polarized before being redirected in the writing area at near-normal incidence.

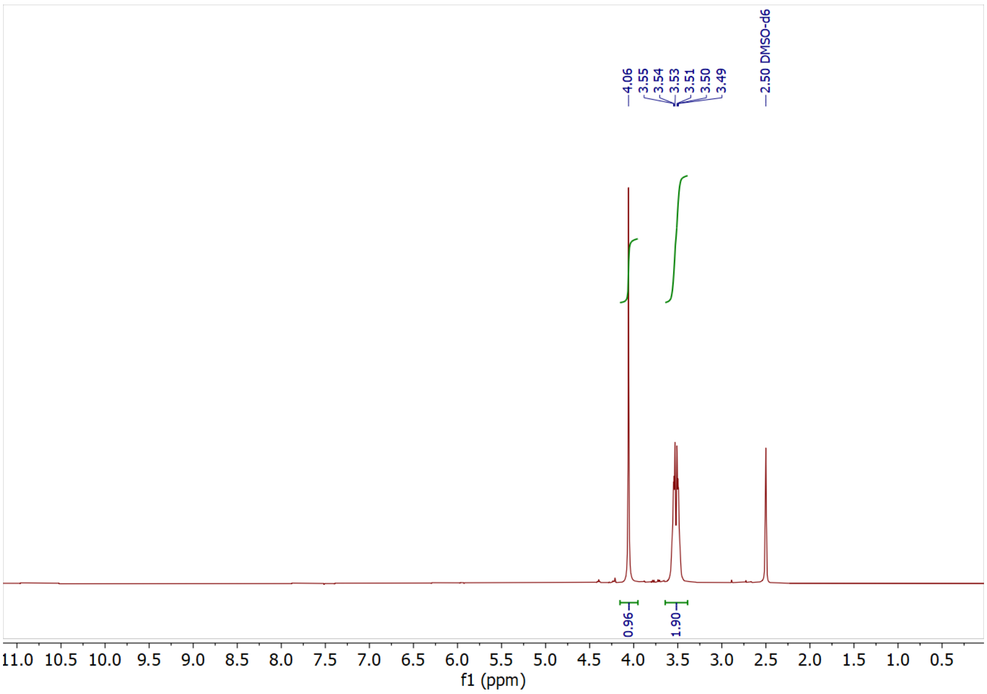

Figure S13 – ^{1}H NMR spectrum of **d**.

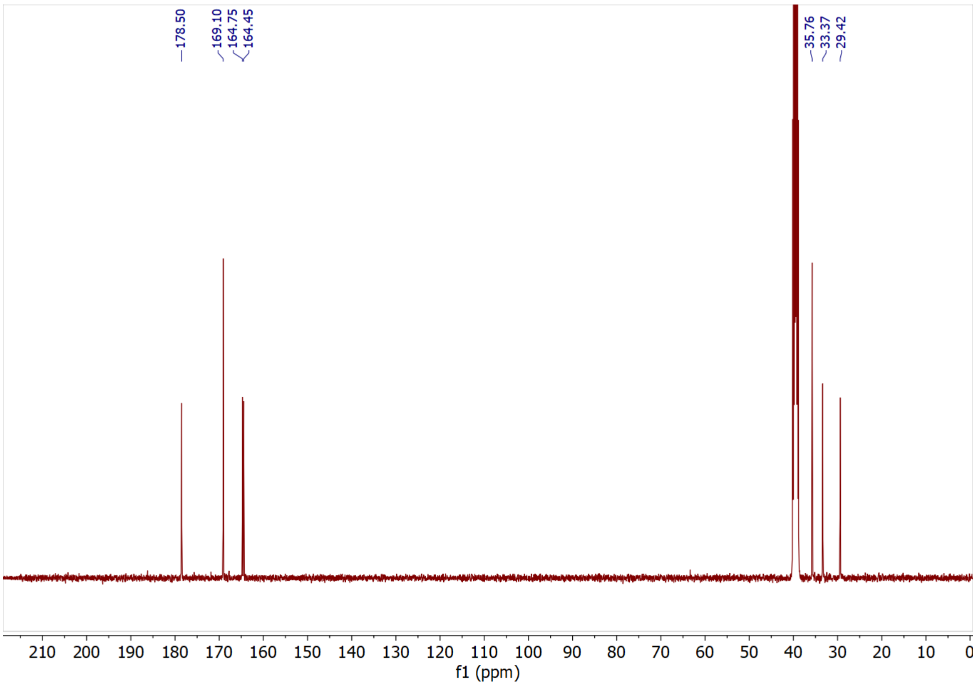

Figure S14 – ^{13}C NMR spectrum of **d**.

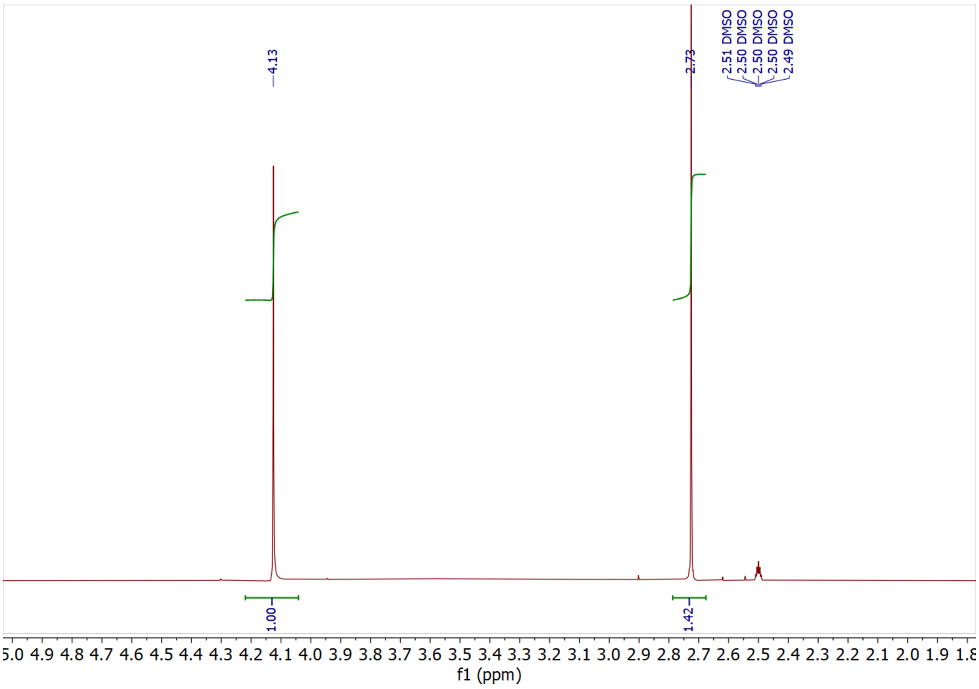

Figure S15 – ^{1}H NMR spectrum of **mc**.

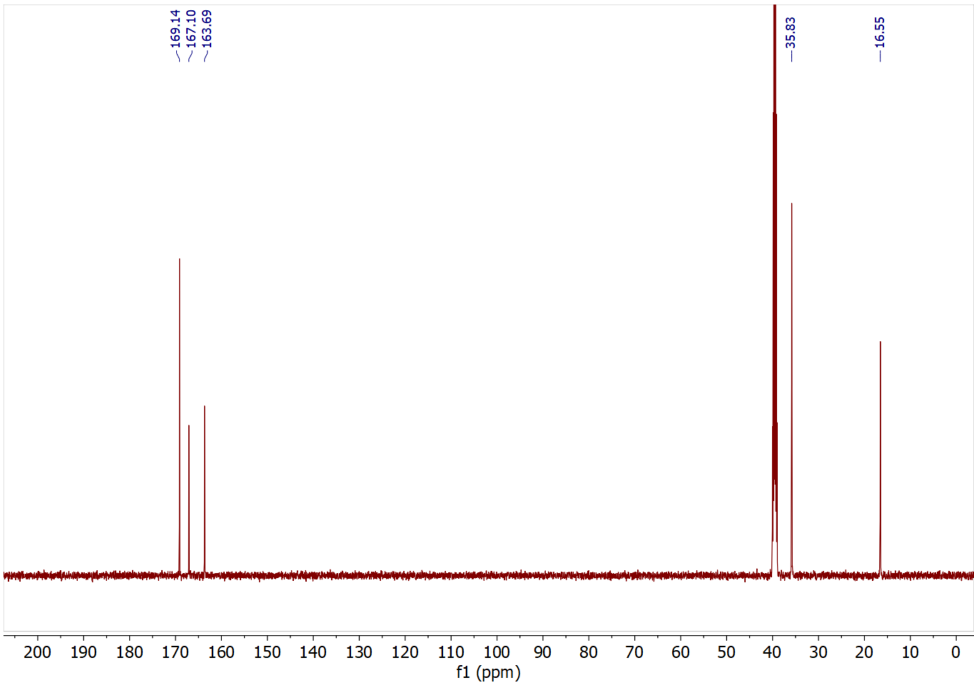

Figure S16 – ^{13}C NMR spectrum of **mc**.

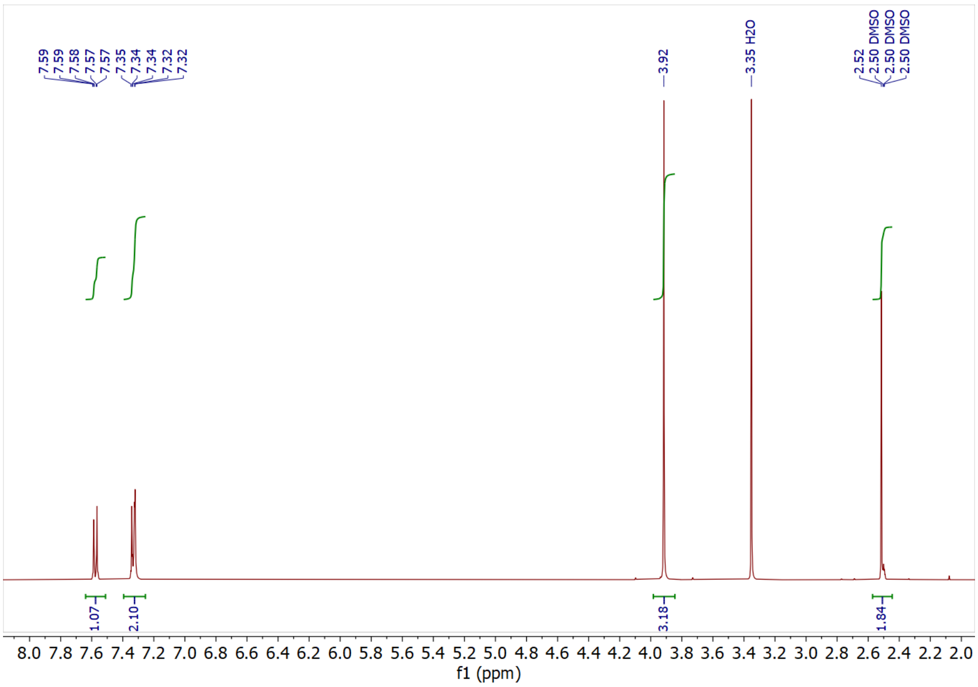

Figure S17 – ^{1}H NMR spectrum of **1**.

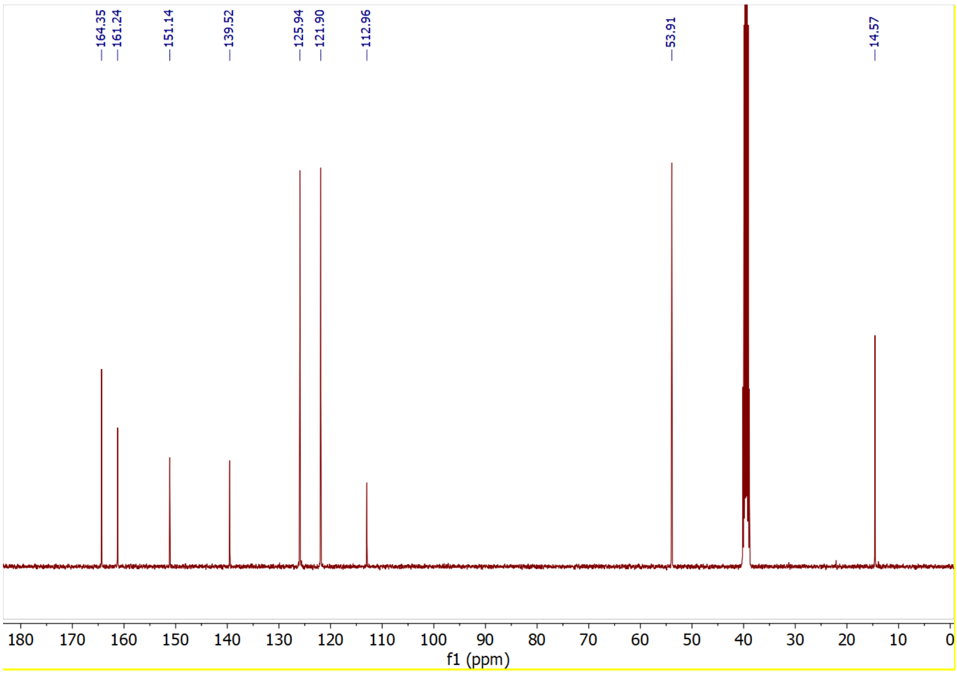

Figure S18 – ^{13}C NMR spectrum of **1**.
